# Supplementary material for: Clinical value of different anti-D immunoglobulin strategies for preventing Rh hemolytic disease of the fetus and newborn: A network meta-analysis
Source: PLoS One. 2020 Mar 12;15(3):e0230073. doi: 10.1371/journal.pone.0230073 (PMC7067404; doi:10.1371/journal.pone.0230073)
Supplement: Attachment — Submitted filename: Response to Reviewers.doc [file pone.0230073.s003.doc]

Dear reviewers:

I am very grateful to your comments for the manuscript. According with your advice, we amended the relevant part in manuscript. Some of your questions were answered below.

--------------------------------------------------------------------

Journal requirements:

1. Please ensure that your manuscript meets PLOS ONE's style requirements, including those for file naming. The PLOS ONE style templates can be found at <http://www.plosone.org/attachments/PLOSOne_formatting_sample_main_body.pdf> and <http://www.plosone.org/attachments/PLOSOne_formatting_sample_title_authors_affiliations.pdf>

**The authors’ answer:** the manuscript has been revised as PLOS ONE's style requirements

2. Please upload a new copy of Figure 2 as the detail is not clear. Please follow the link for more information: [http://blogs.PLOS.org/everyone/2011/05/10/how-to-check-your-manuscript-image-quality-in-editorial-manager/](http://blogs.plos.org/everyone/2011/05/10/how-to-check-your-manuscript-image-quality-in-editorial-manager/)

**The authors’ answer:** The new Figure 2 has been repalced.

3. Please include your tables as part of your main manuscript and remove the individual files. Please note that supplementary tables (should remain/ be uploaded) as separate "supporting information" files

**The authors’ answer:** The tables has been inclued in the manuscript.There is no supplementary tables.

4. We note you have included two different tables in your manuscript labelled as Table 3; 'Table 3 Treatment abbreviations' and 'Table 3 Network meta-analysis result'. Please review these and ensure that you refer to all tables in the text of your manuscript so that they can be separately identified; if accepted, production will need this reference to link the reader to each Table.

**The authors’ answer:** Table label has been modified .Table 3 Treatment abbreviations has been replaced by Table 2 Treatment abbreviations.

Review Comments to the Author

Reviewer #1: The authors investigated the various anti-d immunoglobulin strategies used for prevention of Rh hemolytic disease of the fetus and newborn using a network meta-analysis. Of the nine therapeutic measures studied, they found that giving 300 µg anti-D immunoglobulin at 28 and 34 weeks of gestation was the most effective measure for preventing maternal antibody sensitization, followed by a single injection at 28 weeks of gestation. This is interesting work clearly written.
Reviewer’s comments;
1. Page 3 (Results, last sentence): Suggest adding - Table 2 has the treatment abbreviations.

**The authors’ answer:** The sentence has been added.
2. Page 5 (Table 2): This table was incorrectly labeled as Table 3. Please correct.

**The authors’ answer:** The Table label has been corrected.
3. Page 9 (Discussion, middle of first paragraph): Suggest adding 100 µg to the sentence that states that …then another 500 IU should be administered…

**The authors’ answer:** The “100 µg” has been added in the sentence.
4. Page 9 (Discussion, first paragraph): ACOG is the American College of Obstetricians and Gynecologists. Please correct.

**The authors’ answer:** “American Association of Obstetricians and Gynecologists” has been replaced by “American College of Obstetricians and Gynecologists”.
5. Page 9 (Discussion, first paragraph): The sentence …(ACOG) recommended a single injection of 300 µg anti-D immunoglobulin at 28 weeks of pregnancy with an RhD–positive baby, then another injection of 300 µg again after birth… should be corrected as it is confusing as written. ACOG recommends prophylactic anti-D immune globulin to unsensitized Rh D-negative women at 28 weeks of gestation. After birth, if the baby is Rh D positive, these mothers should receive anti-D immune globulin within 72 hours of birth.

**The authors’ answer:** The confusing sentence has been revised.
6. Page 10 (Discussion, top paragraph, first line): The area in parenthesis after …anti-D antibody positive rates compared with that of the blank/F regimen alone… can be deleted as it already appears in the Results section.

**The authors’ answer:** The area in parenthesis has been deleted.
7. Page 10 (Discussion, top paragraph, line 10): Similarly as in #6 above, the area in parenthesis after …incidence of maternal anti-D positivity…can be deleted.

**The authors’ answer:** The area in parenthesis has been deleted.
8. Page 11 (Discussion, next to last paragraph, last four lines): Please clarify what you mean by …but few studies have focused on the first fetus born to RhD-negative mothers. Supply references of the few studies. Also, please reference the last sentence …Some studies have also found that using anti-D immunoglobulin may cause hemolysis in fetuses during pregnancy.

**The authors’ answer: Confused sentences have been deleted.**

--------------------------------------------------------------------

We acknowledge the reviewer’s comments and suggestions very much, which are valuable in improving the quality of our manuscript.

Sincerely yours,
Xiaohui Xie^1^;Yi Zhang^2^

1.Department of Obstetrics and Gynecology，the First People's Hospital of Neijiang,Neijiang 641000,Sichuan Province, P. R. China2．Department of General Surgery，the First People's Hospital of Neijiang,Neijiang 641000,Sichuan Province, P. R. China

January 30, 2020
