# Supplementary material for: Clinical value of different anti-D immunoglobulin strategies for preventing Rh hemolytic disease of the fetus and newborn: A network meta-analysis
Source: PLoS One. 2020 Mar 12;15(3):e0230073. doi: 10.1371/journal.pone.0230073 (PMC7067404; doi:10.1371/journal.pone.0230073)
Supplement: Attachment — Submitted filename: Response to Reviewers.doc [file pone.0230073.s004.doc]

Dear reviewers:

I am very grateful to your comments for the manuscript. According with your advice, we amended the relevant part in manuscript. Some of your questions were answered below.

--------------------------------------------------------------------

Review Comments to the Author

Reviewer #1: The investigators have addressed previous comments and have made changes to the manuscript. However, I have two additional comments:
1. Results of the Bayesian network meta-analysis (lines 6-7): Based on Table 4, the results for postnatal 3/I vs blank/F should be OR=0.04, 95% CI=0.02-0.06. Please clarify.

Answer: the results for postnatal 3/I vs blank/F has been revised.
2. Figure 5: The labeling of the various curves should be reevaluated. For example, antenatal 5/E had a SUCRA of 96.8%, however the Figure has antenatal 1 with that SUCRA. Antenatal 2/B had a SUCRA of 89.2%, however the Figure has antenatal 3 with that SUCRA.

 Answer:The labeling of the various curves has been reevaluated.The Figure 5 has been replaced.

--------------------------------------------------------------------

We acknowledge the reviewer’s comments and suggestions very much, which are valuable in improving the quality of our manuscript.

Sincerely yours,
Xiaohui Xie^1^;Yi Zhang^2^

1.Department of Obstetrics and Gynecology，the First People's Hospital of Neijiang,Neijiang 641000,Sichuan Province, P. R. China2．Department of General Surgery，the First People's Hospital of Neijiang,Neijiang 641000,Sichuan Province, P. R. China

February 14^th^ 2020
